# Deliberate self-harm and associated risk factors in young adults: the importance of education attainment and sick leave

**DOI:** 10.1007/s00127-020-01893-x

**Published:** 2020-06-16

**Authors:** Ketil Berge Lunde, Lars Mehlum, Ingrid Melle, Ping Qin

**Affiliations:** 1 National Centre for Suicide Research and Prevention, Institute of Clinical Medicine, University of Oslo, Oslo, Norway; 2 NORMENT, K.G. Jebsen Centre for Psychosis Research, Institute of Clinical Medicine, University of Oslo, Oslo, Norway

**Keywords:** Deliberate self-harm, Young adult, Sociodemographic status, Sick leave, Population study

## Abstract

**Purpose:**

The prevalence of deliberate self-harm (DSH) is high in young adults. However, few studies have examined risk in this specific age group. We, therefore, examined the relative influence and interactive nature of a wide range of potential sociodemographic and sick leave related risk factors in young adults, aged 18–35 years, using Norwegian register data.

**Methods:**

All subjects with at least one episode of hospital presentation for DSH registered in the Norwegian Patient Register during the period 2008–2013 were compared with age, gender and date matched population controls using a nested case–control design. The relative influence of factors and their interactions were assessed using conditional logistic regression and recursive partitioning models.

**Results:**

9 873 study cases were compared to 186 092 controls. Socioeconomic status, marital status, sick leave and several demographic factors influenced risk for DSH. Specifically, low education (OR 7.44, 95% CI 6.82–8.12), current sick leave due to psychiatric disorders (OR 18.25, 95% CI 14.97–22.25) and being previously married (OR 3.83, 95% CI 3.37–4.36) showed the highest effect sizes. Importantly, there was an interaction between education and sick leave, where those with either low education and no sick leave (OR 13.33, 95% CI 11.66–15.23) or high education and sick leave (OR 18. 87, 95% CI 17.41–24.21) were the subgroups at highest risk.

**Conclusion:**

DSH in young adults is associated with multiple sociodemographic and health disadvantages. Importantly, the two high-risk subgroups imply different pathways of risk and a need for differentiated preventative efforts.

**Electronic supplementary material:**

The online version of this article (10.1007/s00127-020-01893-x) contains supplementary material, which is available to authorized users.

## Introduction

Young adults are overrepresented among those who present to clinical services after an episode of deliberate self-harm (DSH) [1–3]. As DSH, regardless of underlying motivation or intent, is associated with mental illness and an increased risk for suicide [4], the high rates of DSH among young adults constitute a major public health problem. Preventative efforts are needed and these should be built on a solid understanding of age-specific risk factors; however, few studies on DSH have focused specifically on this age segment of the population.

Young adulthood is characterized by the transition into adult roles and responsibilities [5], where salient developmental tasks include education, workforce entrance and family formation. Failures in these tasks are major sources of distress [6, 7] and can mediate negative effects of pre-existing vulnerabilities [8]. However, most studies on DSH in young adult populations have focused on the influence of distal developmental factors and health problems, for instance childhood adverse experiences, early onset mental problems, educational difficulties and disability [8–13]. Proximal developmental factors have received less attention, despite being proven crucially important for the achievement of adult well-being and mental health [6, 7]. Addressing how such transitions into adulthood influence DSH is, therefore, important to our understanding of risk in the young adult population.

A key challenge when examining these transitional tasks and their association with DSH is that both childhood onset- and emerging adult onset forms of DSH are present in young adults [14]. Importantly, risk factors have been found to differ between those who enter adulthood with pre-existing trajectories of poor functioning, mental health problems and prior DSH, and those who have adult onset of DSH in context of good adolescent adjustment such that distal risk factors, behavioral problems and psychiatric co-morbidity are more common in earlier onset, while reactions to major difficulties in adulthood in context of good adolescent adjustment seems to characterize later onset [15, 16]. This warrants a specific focus on the heterogeneity of risk within young adults, as modelling an average risk across such a diverse population could fail to detect unique high-risk subgroups. An increased knowledge of such subgroups may aid our understanding of different pathways of risk and the development of more targeted preventative efforts.

In this study, we aimed to use the unique source of data from Norwegian national registers to gain more knowledge about DSH among young adults aged 18–35 years. Our specific objectives were: (1) to assess the relative importance of a wide range of sociodemographic and health related variables on risk for DSH and (2) to explore risk factor heterogeneity and specific high-risk subgroups with recursive partitioning model, a method suited to detecting specific high-risk subgroups and risk factor interactions [17].

## Methods

### Data sources

Data were derived from four Norwegian national registers that were interlinked by means of the personal identification number given to all Norwegian residents at birth or immigration. The Central Population Register contains key demographic information including date of birth, sex, link to parents, dating back to 1964. The Norwegian Patient Register contains individual level administrative and medical information on all treatments in Norwegian specialist health care. Data from these registers became person identifiable from 2008. Statistic Norway’s Event Database (FD-Trygd) contains longitudinal information on demographic and socioeconomic factors, including payments of social benefits, dating back to 1992. The Cause of Death register contains the date and cause of death of all Norwegian residents, dating back to 1951.

### Study population and the identification of index episode of DSH

The present study was based on all Norwegian residents aged 18–35 years. From this population we identified all episodes of DSH that received urgent somatic treatment in hospitals and associated services and, therefore, were recorded in *The National Patient Register* during the time period 01.01.2008–31.12.2013. A detailed description of this sampling procedure can be found in a recent publication [18]. In short, we first included all registered treatment episodes, where a Norwegian resident was in contact with specialist health care due to a diagnosis of injury or poisoning according to the International Classification of Diseases (ICD-10: S00-T98 and V0n-Y98). Indirect contacts and planned treatments, fatal injuries and poisoning/injuries that were clearly accidental, inflicted by others or being secondary outcome of other medical conditions were further excluded from our consideration as being ineligible by definition of DSH incident. Due to an underreporting of DSH and use of diagnostic codes for DSH in the Scandinavian administrative health registers [2], we adopted a broader approach to include episodes of probable DSH to prevent detection bias. Based on previous register-based research [19, 20] and our examination of data on incidents which had received a diagnosis of DSH (ICD-code: X6n), three additional steps were taken in a hierarchical fashion to identify probable DSH episodes. The first step was to include treatment contacts because of injuries with a comorbid diagnosis of DSH (ICD-10: X6n, Y87). The second step was to include treatment contacts that had a diagnosis of poisoning (ICD-10: T4n, T50–T55, T57–T60, T62, T62 and T65), open wounds (ICD-10: S10, S11, S15, S17, S19, S21, S25–27, S31, S35–39, S41, S45, S50–51, S54–56, S59, S61, S64–66, S69, S71, S88, T01, T09, T11) or suffocation/drowning or burning (ICD-10: T18, T19, T27–28, T31, T68, T69, T71, T95) and had a comorbid diagnosis of mental or behavioral problems (ICD-10: F0–F9). The final step was to include treatment contacts with poisoning (ICD-10 codes T4n and T50) that were eligible contacts but not covered by the previous steps. After these selection procedures, the first record of contacts by a person was used as the index contact, resulting in a total of 9 873 study cases by young adults of 18–35 years with 3398, 3222 and 3253 cases being derived from the above described three steps, respectively.

### Design

To investigate risk factors for having had a DSH hospital presentation we adopted a nested case–control design [21] to establish a data set comprising all young adults with such a presentation and the population controls for comparison. Each study case was matched with up to 20 population controls randomly selected from a 25% representative sample of the entire Norwegian population with the same sex and birthdate as the case but with no record of treatment contact because of DSH by the calendar time for the index contact of the case in the National Patient Register. With this sampling technique any selected control remains eligible for re-selection as a control and can become a case at a later time if the person presents with the outcome (i.e., DSH). As in many previously published register-based studies on uncommon exposures, we have used up to 20 controls per case to enhance statistical power. This yielded at total of 185 092 controls to be matched for 9 873 cases of young adults at their index or first-recorded episode of DSH.

### Variables of interest

Variables were extracted from the registers by the date of the index presentation of DSH or date of matching. Specifically, socioeconomic factors (i.e., education and income), marital status, sick leave and demographic variables (i.e., ethnicity, area of residence and residential mobility) were derived from the Statistic Norway’s Event Database at the year of index episode or matching. Parental death due to external causes was derived from the Cause of Death Registers.

Education was defined as the highest achieved education at the month prior to the date of the index DSH episode or matching, and classified as: ‘primary’, ‘secondary’, ‘tertiary’ (bachelor, masters or doctoral degree), and’not registered’ educational achievement. A majority of those with missing education information were immigrants (92%). Income was derived from taxable income reported in the year prior to the index episode. Taxable income is recorded in the Norwegian registers in units called the ‘basic amount’ or ‘G’ for short, which are adjusted yearly based on changes in the general income level [22]. During the study period, the basic amount increased from 66 812 NOK to 82 122 NOK and the median income for the adult population was between three and four times the basic amount [23]. Based on this information, income was categorized as: ‘≥4G’, ‘3G’, ‘<3G’ and ‘not registered’. Marital status was classified as: ‘married’, ‘never married’, and ‘previously married’ (i.e., separated, divorced and widowed). Sick leave from work was based on registered paid sick leave, available since the year 1992, categorized as: ‘yes’ and ‘no’. According to the medical cause given in the last sick leave, this variable was further constructed into the following categories: ‘no registered sick leave’, ‘current sick leave due to psychiatric disorders’, ‘current sick leave due to other causes’, ‘prior sick leave due to psychiatric disorders’ and ‘prior sick leave due to other causes’. The classification of cause for sick leave was based on International Classification of Primary Care codes (ICPC-2, codes P01–P99 for psychiatric disorders). Moreover, a variable indicating frequent sick leave (≥ 3 sick leave spells) was constructed for internal comparison. Immigrant status was classified as: ‘native Norwegian’ (having at least one Norwegian born parent) and ‘immigrant’ (both first- and second-generation immigrants). Area of residence was classified according to centrality as defined by Statistics Norway’s [24] into two exclusive categories: ‘central areas’ (the capital area and three largest other cities, i.e., Bergen, Trondheim and Stavanger) and ‘other areas’. Residential mobility was classified as: ‘≤ 2 lifetime residential changes’, ‘3–4 lifetime residential changes’, and ‘≥ 5 lifetime residential changes’. Parental death due to external causes was created by linking cases and controls to their parents using the Central Population Register and then deriving information about parental death and cause of death from the Cause of Death register. Deaths due to external causes were based on the ICD codes E800–E999 from ICD-6/7/8/9 and V01–Y89 from ICD-10. Of the study subjects, 13.2% cases and 14.7% of controls did not have a link to a parent available in the Central Population Register, mostly for being 1st generation immigrants. This variable was then classified into: ‘no’, ‘suicide’ (ICD-6/7: E963, E970–E979; ICD-8/9: E950–E959; ICD-10: X60–X85, Y87.0), ‘other external causes other than suicide’ and ‘no link to parents’.

### Statistical analysis

Conditional logistic regression was used to estimate the associations between DSH and included variables, expressed as odds ratios and 95% confidence intervals. Due to the matched nature of the data set, crude odds ratios were adjusted for age, sex and calendar time. Adjusted odds ratios were further adjusted for all study variables using simultaneous entry, allowing us to control for overlap and correlation between variables. The association differences by sex and age group were examined using the likelihood ratio test. For each variable, a full model including age group or sex interactions for all variables was compared to one, where the interaction was removed for that specific variable.

Recursive partitioning was used to detect specific high-risk subgroups and their risk factor configurations. This multivariable analysis starts by splitting the entire data set into two subgroups based on the strongest predictor of case status. The splitting continues in an iterative manner, selecting the strongest predictor at each new subgroup, creating a set of binary classification rules that can be graphically represented as a classification tree. Splitting continues until no additional variables have been found to improve prediction, creating a terminal node or subgroup. This method is suited to search for higher order interactions as it models risk as a combination of predictors. The most salient interactions detected by the classification tree were then entered into a multivariate conditional logistic regression model.

Two sensitivity analyses were performed to account for potential systematic errors imparted by our sampling procedure. First, we restricted the primary analysis to study cases that were sampled with a diagnosis of ICD-10 X6n to assess possible deviations of findings due to case inclusion. Second, we restricted the primary analysis only to study cases included in the last year of our 6-year study period to ensure that they had at least 5-year observation time within the data coverage period and that their index episode was more likely the first episode of DSH.

Retrieval of data from source registers and construction of data set for analysis were conducted using SAS/STAT software, version 9.4 of the SAS System for Windows [25]. All statistical analyses were conducted with the R statistical software, version 3.4.4 [26]. Specifically, the software package “survival” version 2.38 [27] was used to perform conditional logistic regression and “rpart” version 4.1-15 [28] was used to model the classification tree.

## Results

### Sample characteristics

**Fig. 1:**
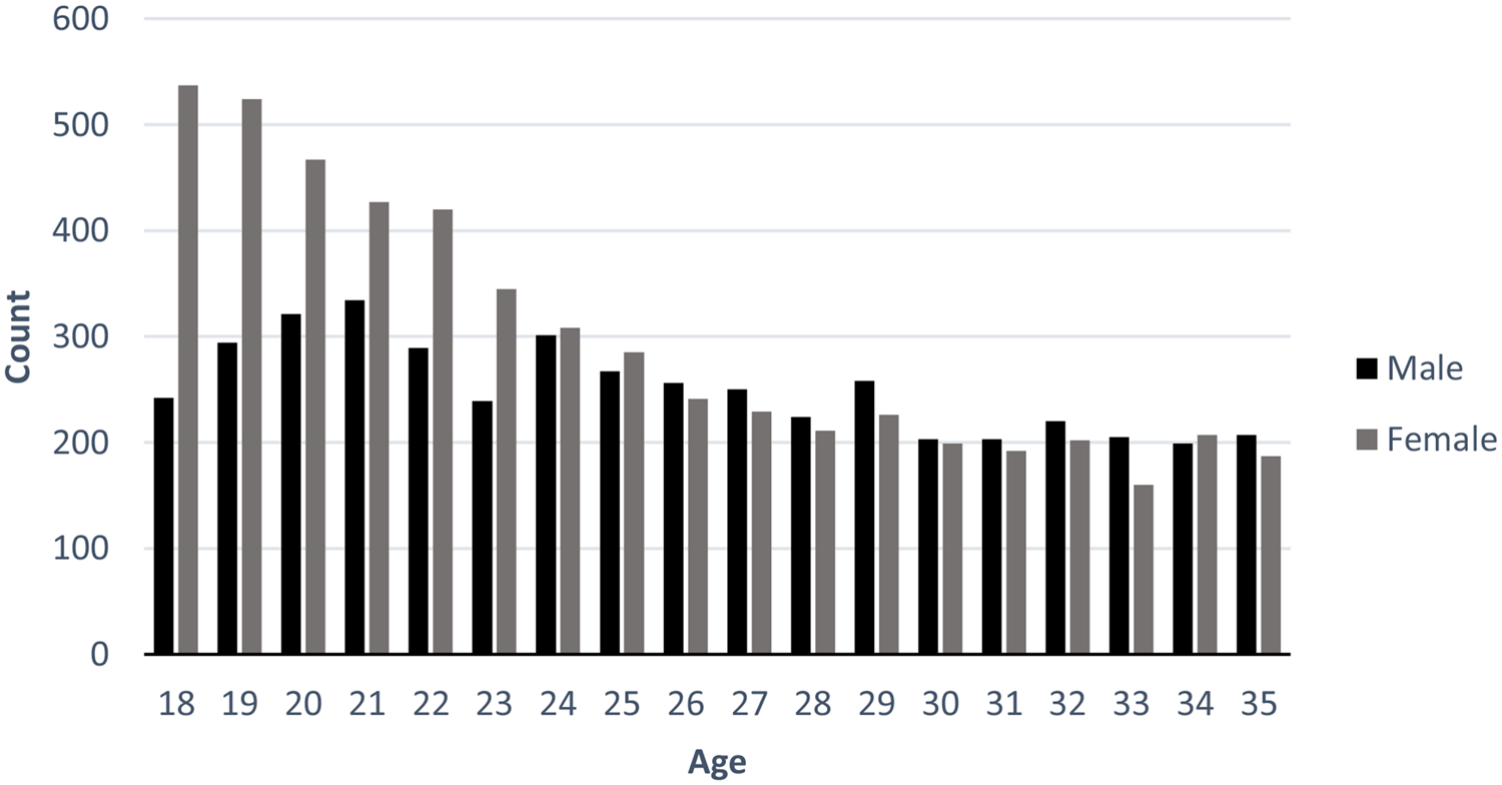
Age distribution of study cases by sex

Of the 9873 study cases, 5367 (54.4%) were female and 4506 (45.6%) were male. The age distribution of study cases is shown for females and males in Fig. 1. It is evident that females were overrepresented in the early part of young adulthood and that the gender distribution was about equal for the age group 25–35 years. This was also reflected in the mean age being lower for female cases (24.6, SD 5.1) than male cases (25.8, SD 5.1).

### Risk factors for DSH

The distribution of study variables compared for study cases and controls is shown in Table 1. The cases were significantly more often disadvantaged in terms of socioeconomic status, marital status, sick leave, residential mobility and loss of parents compared to population controls. Results from both univariate- and multivariate conditional logistic regression, reported as crude and adjusted odds ratios, are also shown in Table 1. All the variables were associated with DSH in the univariate conditional logistic regression (crude ORs). The magnitude of associations was generally attenuated in the multivariate analysis (adjusted ORs); however, all variables remained significantly associated with DSH. Importantly, the highest odds ratios in the adjusted analysis were found for low education, sick leave, marital dissolution and low income.

**Table 1:** Distribution of study variables and associated risks for deliberate self-harm

	Distribution, N (%)		Risk factors for deliberate self-harm	
	Cases	Controls	Unadjusted^a^	Adjusted^b^
	1 (*n* = 9873)	0 (*n* = 185,092)	OR (95% CI)	OR (95% CI)
*Education level*				
Tertiary	675 (6.8)	47,240 (25.5)	1.00 (Reference)	1.00 (Reference)
Secondary	2389 (24.2)	70,197 (37.9)	2.83 (2.59–3.09)	2.31 (2.11–2.53)
Primary	6342 (64.2)	57,788 (31.2)	11.10 (10.22–12.07)	7.44 (6.82–8.12)
Unknown	467 (4.7)	9867 (5.3)	3.72 (3.30–4.20)	4.96 (4.30–5.71)
*Income level*				
≥ 4G	1755 (17.8)	60,348 (32.6)	1.00 (Reference)	1.00 (Reference)
3G	1515 (15.3)	21,003 (11.3)	2.89 (2.69–3.11)	1.90 (1.76–2.05)
< 3G	6493 (65.8)	99,848 (53.9)	3.14 (2.95–3.34)	2.86 (2.68–3.06)
Not registered	110 (1.1)	3893 (2.1)	1.13 (0.93–1.38)	1.59 (1.29–1.97)
*Marital status*				
Married	661 (6.7)	28,259 (15.3)	1.00 (Reference)	1.00 (Reference)
Never married	8670 (87.8)	153,303 (82.8)	2.62 (2.41–2.85)	2.26 (2.06–2.47)
Previously married	542 (5.5)	3530 (1.9)	6.42 (5.70–7.23)	3.83 (3.37–4.36)
*Sick leave*				
No	5352 (54.2)	127,468 (68.9)	1.00 (Reference)	–
Yes	4521 (45.8)	57,624 (31.1)	2.22 (2.12–2.33)	–
*Sick leave by cause*				
No record	5352 (54.2)	127,468 (68.9)	–	1.00 (Reference)
Current psychiatric	251 (2.5)	333 (0.2)	–	18.25 (14.97–22.25)
Current other	943 (9.6)	6113 (3.3)	–	3.90 (3.57–4.26)
Prior psychiatric	709 (7.2)	2947 (1.6)	–	3.77 (3.38–4.21)
Prior other	2618 (26.5)	48,231 (26.1)	–	1.28 (1.21–1.36)
*Sick leave spells*^c^				
3 or more	2108 (21.4)	21,326 (11.5)	–	1.16 (1.08–1.24)
*Immigrant*				
Norwegian	8534 (86.4)	152,867 (82.6)	1.00 (Reference)	1.00 (Reference)
Immigrant	1339 (13.6)	32,225 (17.4)	0.74 (0.69–0.78)	0.86 (0.78–0.94)
*Area of residence*				
Rural	8316 (84.2)	158,008 (85.4)	1.00 (Reference)	1.00 (Reference)
Central	1557 (15.8)	27,084 (14.6)	1.09 (1.03–1.16)	1.25 (1.18–1.32)
*Residential mobility*				
≤ 2 residential changes	5301 (53.7)	132,274 (71.5)	1.00 (Reference)	1.00 (Reference)
3-4 residential changes	2553 (25.9)	38,414 (20.8)	1.72 (1.63–1.80)	1.53 (1.46–1.61)
≥5 residential changes	2019 (20.4)	14,404 (7.8)	3.65 (3.45–3.86)	2.28 (2.15–2.42)
*Parental sudden death*				
No record	8568 (86.8)	157,904 (85.3)	1.00 (Reference)	1.00 (Reference)
Suicide	156 (1.6)	925 (0.5)	3.10 (2.61–3.68)	2.08 (1.72–2.52)
Other causes	144 (1.5)	1309 (0.7)	2.04 (1.72–2.43)	1.40 (1.16–1.69)
No link	1005 (10.2)	24,954 (13.5)	0.73 (0.68–0.78)	0.86 (0.77–0.96)

^a^Crude ORs were adjusted for age, gender and calendar time through matching

^b^Adjusted ORs were further adjusted for all included variables

^c^Internal comparison, ORs representing added effect of respective variable for those with a registered sick leave

Specifically, compared to those with the highest education level, risk increased progressively with decreasing education attainment from the secondary (OR 2.31, 95% CI 2.11–2.53, in the adjusted model) to the primary education levels (OR 7.44, 95% CI 6.82–8.12). A pattern of progressively increased risk with lower income was also evident, although not as steep as for education (3G: OR 1.90, 95% CI 1.76–2.05; > 3G: OR 2.86, 95% CI 2.68–3.06). For sick leave, a prior sick leave spell was a strong risk factor if it was due to a psychiatric disorder (OR 3.77, 95% CI 3.38–4.21). Moreover, risk increased substantially for those who were currently on sick leave, regardless of cause, but especially if the sick leave was due to psychiatric disorders (current psychiatric sick leave: 18.25, 95% CI 14.97–22.25; current other cause: OR 3.90, 95% CI 3.57–4.26). There was a small additional increase in risk for those who had had 3 or more sick leave spells. Finally, there was an increased risk of being single compared to being married, especially for those who were separated, divorced or widowed (OR 3.83, 95% CI 3.37–4.36). As for the remaining factors, it was evident that loss of parents due to external causes of death, living in urban compared to rural areas and high residential mobility were associated with increased risk of DSH. Being non-Norwegians, however, was associated with a reduced risk for DSH compared to being native-Norwegian counterparts.

### Age and gender differences in risk factors for DSH

Adjusted odds ratios and results from the interaction tests are shown separately for the age group (18–24 years and 25–35 years) and sex stratified analyses in Table 2. Across both stratifications, the direction of associations was the same. However, the interaction tests indicate that age group and sex influenced the magnitude of effect size in varying degrees for most of the included variables.

**Table 2:** Distribution of study variables and associated risks for deliberate self-harm, stratified by age group and by sex

	Analysis by age group					Analysis by sex				
	*N* (Case/control)		Adjusted odds ratios (95% CI)			*N* (Case/control)		Adjusted Odds ratios (95% CI)		
	18–24 years	25–35 years	18–24 years	25–35 years	Interaction	Male	Female	Male	Female	Interaction
*Education*										
Tertiary	109/10,447	566/36,793	1.00 (Reference)	1.00 (Reference)	82.87**	171/18,628	504/28,612	1.00 (Reference)	1.00 (Reference)	39.10**
Secondary	1052/40,016	1337/30,181	2.82 (2.30–3.47)	2.41 (2.18–2.68)		970/34,409	1419/35,788	2.92 (2.46–3.45)	2.31 (2.11–2.53)	
Primary	3694/40,166	2648/17,622	11.00 (8.99–13.45)	6.17 (5.59–6.81)		3154/27,166	3188/30,622	10.24 (8.71–12.04)	7.44 (6.82–8.12)	
Unknown	193/3855	274/6012	8.25 (6.35–10.73)	3.81 (3.19–4.56)		211/4837	256/5030	8.25 (6.51–10.45)	4.96 (4.30–5.71)	
*Income*										
≥ 4G	366/8965	1389/51,383	1.00 (Reference)	1.00 (Reference)	83.43**	980/36,379	775/23,969	1.00 (Reference)	1.00 (Reference)	47.56**
3G	527/8331	988/12,672	1.32 (1.14–1.53)	2.08 (1.90–2.28)		665/8445	850/12,558	2.30 (2.06–2.57)	1.90 (1.76–2.05)	
< 3G	4111/75,539	2382/24,309	1.76 (1.56–2.00)	3.54 (3.28–3.83)		2816/38,368	3677/61,480	3.55 (3.24–3.89)	2.86 (2.68–3.06)	
Not registered	44/1649	66/2244	1.01 (0.72–1.42)	1.78 (1.36–2.33)		45/1848	65/2045	1.82 (1.31–2.53)	1.59 (1.29–1.97)	
*Marital status*										
Married	97/2516	564/25,743	1.00 (Reference)	1.00 (Reference)	8.08*	191/11,625	470/16,634	1.00 (Reference)	1.00 (Reference)	7.10*
Never married	4911/91,805	3759/61,498	1.65 (1.32–2.06)	2.34 (2.12–2.59)		4151/72,195	4519/81,108	2.46 (2.09–2.89)	2.26 (2.06–2.47)	
Previously married	40/163	502/3367	3.50 (2.25–5.42)	4.14 (3.62–4.74)		164/1220	378/2310	3.43 (2.71–4.35)	3.83 (3.37–4.36)	
*Sick leave by cause*										
No record	3618/81,134	1734/46,334	1.00 (Reference)	1.00 (Reference)	47.62**	2349/60,737	3003/66,731	1.00 (Reference)	1.00 (Reference)	40.93**
Current psychiatric	102/42	149/291	47.15 (31.75–70.02)	11.49 (9.00–14.67)		98/121	153/212	13.74 (9.92–19.03)	18.25 (14.97–22.25)	
Current other	443/1855	500/4258	4.26 (3.76–4.83)	3.27 (2.87–3.71)		428/1900	515/4213	5.29 (4.61–6.07)	3.90 (3.57–4.26)	
Prior psychiatric	84/177	625/2770	6.79 (5.05–9.13)	3.10 (2.74–3.50)		353/1324	356/1623	3.34 (2.85–3.92)	3.77 (3.38–4.21)	
Prior other	801/11,276	1817/36,955	1.28 (1.17–1.40)	1.16 (1.07–1.26)		1278/20,958	1340/27,273	1.41 (1.29–1.54)	1.28 (1.21–1.36)	
*Sick leave spells*^a^										
3 or more	357/2206	1751/19,120	1.16 (1.01–1.34)	1.24 (1.14–1.35)	0.61	1026/7677	1082/13,649	1.42 (1.28–1.58)	1.16 (1.08–1.24)	29.48**
*Immigrant status*										
Norwegian	4445/81,958	4089/70,909	1.00 (Reference)	1.00 (Reference)	7.90**	4021/70,111	4513/82,756	1.00 (Reference)	1.00 (Reference)	39.98**
Immigrant	603/12,526	736/19,699	0.94 (0.84–1.06)	0.72 (0.61–0.84)		485/14,929	854/17,296	0.59 (0.51–0.69)	0.86 (0.78–0.94)	
*Residential mobiltiy*										
≤ 2 residential changes	2986/73,995	2315/58,279	1.00 (Reference)	1.00 (Reference)	29.30**	2418/62,091	2883/70,183	1.00 (Reference)	1.00 (Reference)	3.31
3–4 residential changes	1223/15,148	1330/23,266	1.68 (1.56–1.80)	1.35 (1.25–1.45)		1181/16,950	1372/21,464	1.62 (1.50–1.75)	1.53 (1.46–1.61)	
≥ 5 residential changes	839/5341	1180/9063	2.60 (2.38–2.85)	1.95 (1.80–2.12)		907/5999	1112/8405	2.41 (2.20–2.64)	2.28 (2.15–2.42)	

Area of residence’ and ‘Parental death due to external causes’ is not included in table due to no significant interaction with age group and sex

*Interaction test *p* value < 0.05, **Interaction test *p* value < 0.01

^a^Internal comparison, ORs representing the added effect of respective variable for those with history of sick leave

When comparing the two age groups, a higher risk associated with low education (Test of interaction: *x*^2^ = 82.87, *p* < 0.001) and sick leave (*x*^2^ = 47.62, *p* < 0.001) were seen in the young aged 18–24 years than seen in those aged 25–35 years. Low income was also a stronger risk factors among those aged 25–35 years (*x*^2^ = 83.43, *p* < 0.001). When looking into sex differences, it was evident that both measures of socioeconomic disadvantage were associated with a higher risk of DSH in men than that in women (Education level: x^2^ = 39.10, *p* < 0.001; Income level: *x*^2^ = 47.55, *p* < 0.001), while status as immigrant seemed to be more protective against DSH for men than for women (*x*^2^ = 39.98, *p* < 0.001). Sex also significantly differentiated the effect estimate of sick leave (*x*^2^ = 40.93, *p* < 0.001), with a stronger effect of sick leave due to psychiatric disorders in women and a stronger effect of sick leave due to other causes in men.

### High-risk subgroups

**Fig. 2:**
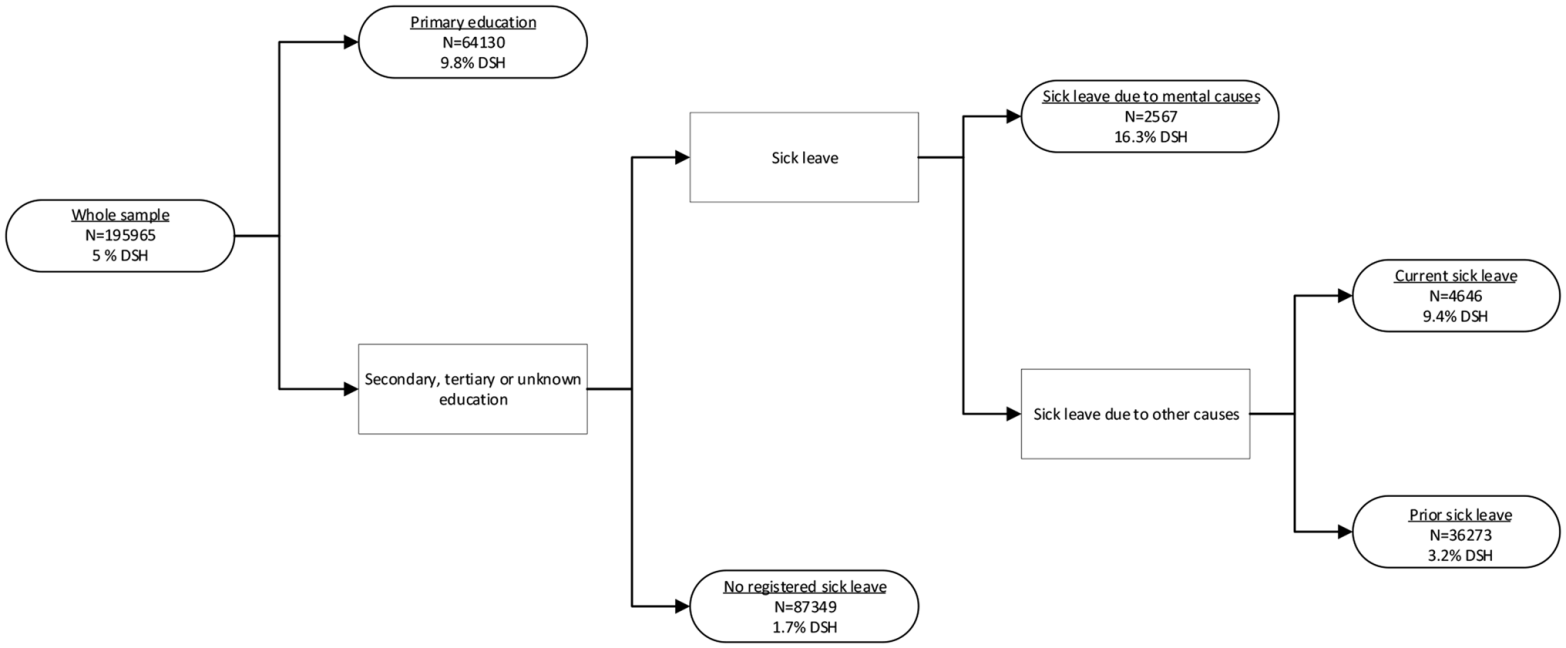
Classification tree

The classification tree produced by recursive partitioning model can be seen in Fig. 2. Of all the variables included in the model, the classification tree analysis found education to be the most important predictor of case status with primary education as the high-risk cutoff. In the primary education subgroups, no additional factors were found to improve prediction. Further splits were, however, found in the subgroup containing those with secondary, tertiary or unknown education. Specifically, two high-risk subgroups were found in this higher educated population: those with a history of sick leave spell(s) due to a psychiatric disorder; and those with a current sick leave due to a non-psychiatric disorder were found to be two high-risk groups.

Based on these findings, we included the interaction between sick leave and education in a conditional logistic regression analysis. For this analysis, the sick leave variable was collapsed to ensure sufficient statistical power, into: no sick leave (reference); sick leave due to a psychiatric disorder and sick leave due to other causes. Results of this analysis, adjusted for all the other factors, are shown in Table 3. Here we see that the risk associated with having had a sick leave spell increased with higher education. This was especially evident for sick leave due to a psychiatric disorder (Primary: OR 3.37, 95% CI 2.98–3.80; Secondary: OR 9.25, 95% CI 7.94–10.77; Tertiary: OR 18.87, 95% CI 17.41–24.21). In addition, the effect size of primary education increased substantially when this interaction term was entered into the model (OR 13.33, 95% CI 11.66–15.23).

**Table 3:** Interactive influence between education and sick leave

	Distribution	Risk for deliberate self-harm
	*N* (cases/controls)	Adjusted OR^a^ (95% CI)
*Education*		
Tertiary	251/31,451	1.00 (Reference)
Secondary	944/45,755	2.73 (2.37–3.15)
Primary	3884/41,637	13.33 (11.66–15.23)
Not registered	323/8625	6.65 (5.54–7.98)
*Sick leave due to psychiatric disorder, by education*^b^		
Tertiary	106/778	18.87 (17.41–24.21)
Secondary	297/1336	9.25 (7.94–10.77)
Primary	541/1132	3.37 (2.98–3.80)
Not registered	16/34	8.83 (4.37–16.05)
*Sick leave due to other causes, by education*^b^		
Tertiary	318/15,011	3.45 (2.91–4.10)
Secondary	1148/23,106	2.59 (2.36–2.85)
Primary	1967/15,019	1.18 (1.11–1.26)
Not registered	128/1208	3.14 (2.51–3.93)

^a^Adjusted for age, gender, calendar time, income, marital status, ethnicity, area of residence, residential mobility and parental death due to external causes

^b^Log-likelihood ratio test of interaction: *x*^2^ = 416.77, *p* < 0.001

### Sensitivity analyses

Results were almost identical with the main analysis when we restricted the analysis to both study cases with a registered diagnosis of ICD-10 X6n and study cases with a 5-year observation period prior to the index event (see Online Resource 1). The magnitude of effect sizes varied slightly, especially for the relatively uncommon categories of sick leave and immigrant status, which could possibly be the result of reduced sample sizes.

## Discussion

The present study used Norwegian national register data to gain insights into young adults who presented to hospitals and associated services for somatic treatment because of DSH. The study is to our awareness the first to combine conditional logistic regression and recursive partitioning to illustrate the relative influence and interactive effects of a wide range of sociodemographic and health factors on risk for DSH. It demonstrated the influence of multiple risk factors that have previously been established in adolescents and all adults, including socioeconomic status [29], marital status [30], sick leave [31], being native Norwegian [32], living in urban areas [33], residential mobility [34] and loss of parents due to external deaths [35]. Of these, low education attainment, a history of sick leave due to psychiatric disorders, a current sick leave spell and being previously married revealed the highest effect sizes in the adjusted analyses, implying that these characteristics constitute especially salient indicators of risk for DSH in this age band. Importantly, education served as an effect modifier for the risk associated with sick leave, alluding to two unique high-risk subgroups.

The high risks associated with socioeconomic disadvantage in general and education in particular add further support to the strong socioeconomic gradient in DSH among young people [12, 29]. Similarly, the more pronounced risk associated with socioeconomic disadvantage in men than in women is also in line with findings from previous studies [36]. This association could be due to fewer coping skills and less resources available for help in times of crisis among those with lower education [37]. Low education could also make it more to enter the workforce, compete for jobs and achieve financial independence, crucial developmental tasks for young adults in western societies [6, 37]. Importantly, the strong effect size of the lowest education level on DSH may reflect the confounding from distal factors, as educational problems in adolescence are known proxies for cognitive abilities [37, 38] and childhood mental health problems [39]. While our study cannot delineate the exact mechanisms in which education influence the risk for DSH, an interplay between both distal and proximal developmental factors is likely present, where early life difficulties create pathways of cascading developmental failures [40, 41].

As for the strong influence of sick leave, the most obvious explanation could be the underlying health problems that lead to the sick leave spell, with mental illness especially common among young patients with DSH [42]. Interestingly, we found that sick leave due to a psychiatric disorder constituted a stronger risk factor for DSH in the age group of 18–24 years than for the group of 25–35 years. In Norway, it is common form young people aged 18–24 years to undertake education, vocational training or to be at an early stage of establishing their career. Sick leave at this age, therefore, implies a risk of a delay or discontinuation of these important tasks, which in turn can increase the likelihood of disability, social isolation and socioeconomic marginalization [43, 44].

Results from the classification tree analysis lend further support to the importance of education and sick leave, as these were the two factors selected as predictors of DSH. Importantly, our results suggest that the risk associated with sick leave increased substantially for young adults with higher education. This was evident for both past and ongoing sick leave due to psychiatric and ongoing sick leave due to other disorders. Differences in risk factors for DSH between education subgroups have been documented [45, 46] and, specifically, an interaction between education and sick leave on risk for suicide have been documented with Norwegian register data [47]. One explanation for this interaction could be that loss of ability due to health problems, regardless of disorder, represent a greater stressor for the highest educated due to greater career aspirations and demands. Interestingly, we also found an increased risk of DSH associated with having primary education in the context of no history of sick leave. As employment is a requirement for paid sick leave in Norway, the high risk seen for those with lower education in context of no registered sick leave could represent confounding by psychiatric disorders associated with a high degree of self-selection into low qualifications and unemployment, such as early onset substance abuse, psychosis and personality disorders. This group could, therefore, be conceptualized with a continuation of poor adaptation from adolescence to adulthood, while those with higher education presumably have negotiated many of the developmental challenges of adolescence and early adulthood, including entering the workforce. Key differences between the two pathways may, therefore, center on differences in age of onset of psychiatric disorders and the degree of self-selection [41, 46]. However, an important commonality is that both pathways include occupational difficulties in some form. In turn, this highlights the importance of difficulties or failures in the domain of work accomplishments on risk for DSH in this young population.

While it was evident that education and work accomplishments were strongly associated with risk for DSH, the influence of relational factors were more uncertain. In line with the previous studies on all adults we found that not being married, especially after marital dissolution, was associated with increased risk in our sample [30, 48]. However, there are several caveats regarding marital status in this population that needs to be taken into account when interpreting these findings. First, there were relatively few married in the youngest age group. Second, due to limited information in our data source, cohabitation status was included as single category in the analysis. Since cohabitation is a common family form in Norway and has the same protective effect as marriage [48, 49], this could bias the effect size of being single towards the null. Overall, while marriage has a protective effect in our analysis, our results suggest that its contribution to risk for DSH in young adults seem to be less important compared to the middle aged, which conforms to previous findings [50].

Finally, we found that immigrant status was associated with reduced risk of DSH, which aligns well with a recent study on suicide among immigrants in Norway [32]. Two possible explanations for this association include that immigrants from countries with lower rates of DSH and suicide maintain this lower rate after immigration to Norway [51] and that immigrants may represent a relatively healthy part of the population in the country of origin [52]. It should be noted that our immigration variable was crude and may, therefore, mask subgroup differences in risk for DSH among immigrants.

## Strengths and limitations

Several limitations should be taken into account when interpreting the results from this study. First, index episodes of DSH were not necessarily the first DSH episode in the study population as individual level data from the Norwegian Patient Register were only available from 01.01.2008. Second, the episodes of DSH identified through our algorithm included both diagnosed and probable episodes of DSH. This was done on the basis of considerable underreporting of DSH in the administrative registers [2]. Including probable episodes of DSH may introduce a source of detection bias, as a proportion of these injuries can be unintentional. However, in line with previous studies using register data [2, 53], underreporting of DSH was deemed a more serious threat to the validity of our results [18]. Moreover, register data are collected for an administrative purpose, which is limiting the type of information available. Well-established risk factors such as mental illness, adverse childhood experiences, recent stressful life events and relational factors other than marital status [4] could not be included in the analysis, hence, these can act as residual confounders. This will consequently lead to a bias towards overestimating the effect sizes.

With the above limitations in mind, the study also has important strengths. Data from national registers allow for large sample sizes, making it possible to examine the association between rare outcomes and many variables simultaneously with strong statistical power. In addition, as there is no need to actively recruit participants which eliminates the risk for selection bias. Information in the registers is collected longitudinally and systematically for the entire national population, reducing the risk of differential misclassification bias. In addition, the inclusion of recursive partitioning as a method to elucidate possible interactions and etiological differences is an important strength. Compared to other data-driven methods of interaction detection, such as stepwise regression, recursive partitioning offers a transparent, graphic and easy to understand description of interactive structures in the data set that is similar to the way clinicians think about risk and less susceptible to loss of power and collinearity [55].

## Conclusions and implications

Our study added to existing literature by quantifying the relative influence and interactive nature of a wide range of risk factors on DSH in a large and representative sample of young adults. Importantly, our results give added support to developmental subtypes of risk and highlights the need for an increased focus on the interactive nature of risk factors, which to date have received less attention [56].

As for clinical implications, our study indicates that education plays a crucial role as a risk factor for hospital presented DSH in this young adult population. Moreover, the two distinct risk profiles found could have implications for when and to whom preventative efforts should be targeted. The most common of these pathways was characterized by low education. Early school based efforts to detect mental health problems interfering with educational attainment may, therefore, be an appropriate preventative strategy to address negative trajectories of socioeconomic disadvantage and potentially also DSH in young adults. Moreover, this could be an especially important when targeting DSH in males. The sociodemographic factors were less discriminant in the higher educated, where sick leave seems to delineate high-risk groups best. This implies the occurrence of different pathways towards DSH that might be more difficult to detect and target with early preventative efforts from a public health perspective. For this group, ensuring that general practitioners consider symptoms of mental illness and the need for mental health treatment as part of awarding sick leave spells in this young population could be an appropriate strategy to ensure that at-risk young adults are detected.

## Electronic supplementary material

Below is the link to the electronic supplementary material.

Supplementary material 1 (DOCX 21 kb)
