# Supplementary material for: Deliberate self-harm and associated risk factors in young adults: the importance of education attainment and sick leave
Source: Soc Psychiatry Psychiatr Epidemiol. 2020 Jun 16;56(1):153–64. doi: 10.1007/s00127-020-01893-x (PMC7847451; doi:10.1007/s00127-020-01893-x)
Supplement: Supplementary file 1 — Supplementary material 1 (DOCX 21 kb) [file 127_2020_1893_MOESM1_ESM.docx]

Supplementary material: Sensitivity analyses

|  | **Only study cases with diagnosed DSH (ICD-10 X6n)** | | | **Only subjects sampled in 2013** | | |
| --- | --- | --- | --- | --- | --- | --- |
|  | **Distribution, N (%)** | | **Risk factors** | **Distribution, N (%)** | | **Risk factors** |
|  | **Cases** | **Controls** | **Adjusted** | **Cases** | **Controls** | **Adjusted** |
|  | **1 (n=3398)** | **0 (n=63680)** | **OR (95% CI)** | **1 (n=1358)** | **0 (n=26032)** | **OR (95% CI)** |
| **Education** |  |  |  |  |  |  |
| Tertiary | 271 (8.0) | 16351 (25.7) | 1.00 (Reference) | 91 (6.7) | 6116 (23.5) | 1.00 (Reference) |
| Secondary | 876 (25.8) | 23756 (37.3) | 2.16 (1.86-2.50) | 297 (21.9) | 8913 (34.2) | 2.17 (1.69-2.77) |
| Primary | 2075 (61.1) | 20237 (31.8) | 6.36 (5.52-7.33) | 891 (65.6) | 9191 (35.4) | 6.67 (5.27-8.45) |
| Unknown | 176 (5.2) | 3336 (5.2) | 4.27 (3.39-5.39) | 79 (5.8) | 1812 (7.0) | 4.20 (2.91-6.07) |
| **Income** |  |  |  |  |  |  |
| ≥4G | 634 (18.7) | 19556 (30.7) | 1.00 (Reference) | 203 (14.9) | 7561 (29.0) | 1.00 (Reference) |
| 3G | 520 (15.3) | 7314 (11.5) | 1.76 (1.54-2.01) | 197 (14.5) | 2872 (11.0) | 1.94 (1.56-2.41) |
| <3G | 2197 (64.7) | 35528 (55.8) | 2.48 (2.21-2.79) | 947 (69.7) | 15276 (58.7) | 2.90 (2.40-3.49) |
| Not registered | 47 (1.4) | 1282 (2.0) | 1.79 (1.28-2.50) | 11 (0.8) | 323 (1.2) | 1.81 (0.92-3.56) |
| **Marital status** |  |  |  |  |  |  |
| Married | 263 (7.7) | 9564 (15.0) | 1.00 (Reference) | 79 (5.8) | 3615 (13.9) | 1.00 (Reference) |
| Never married | 2926 (86.1) | 52846 (83.0) | 1.92 (1.66-2.23) | 1224 (90.1) | 21989 (84.5) | 2.44 (1.88-3.15) |
| Previously married | 209 (6.2) | 1270 (2.0) | 3.76 (3.04-4.64) | 55 (4.1) | 428 (1.6) | 3.32 (2.27-4.86) |
| **Sick leave by cause** |  |  |  |  |  |  |
| No record | 1828 (53.8) | 43852 (68.9) | 1.00 (Reference) | 786 (57.9) | 18697 (71.8) | 1.00 (Reference) |
| Current psychiatric | 101 (3.0) | 134 (0.2) | 20.39 (14.85-27.99) | 20 (1.5) | 50 (0.2) | 9.80 (5.35-17.93) |
| Current other | 363 (10.7) | 2162 (3.4) | 4.43 (3.81-5.14) | 164 (12.1) | 1592 (6.1) | 3.19 (2.58-3.95) |
| Prior psychiatric | 234 (6.9) | 997 (1.6) | 4.20 (3.47-5.08) | 52 (3.8) | 232 (0.9) | 3.24 (2.23-4.71) |
| Prior other | 872 (25.7) | 16535 (26.0) | 1.34 (1.21-1.49) | 336 (24.7) | 5461 (21.0) | 1.50 (1.27-1.78) |
| **Sick leave spells^1^** |  |  |  |  |  |  |
| 3 or more | 719 (21.2) | 7484 (11.8) | 1.08 (0.95-1.22) | 243 (17.9) | 2708 (10.4) | 1.02 (0.84-1.24) |
| **Immigrant** |  |  |  |  |  |  |
| Norwegian | 2873 (84.5) | 52739 (82.0) | 1.00 (Reference) | 1138 (83.8) | 20766 (79.8) | 1.00 (Reference) |
| Immigrant | 525 (15.5) | 10941 (17.2) | 1.03 (0.89-1.20) | 220 (16.2) | 5266 (20.2) | 0.98 (0.78-1.24) |
| **Area of residence** |  |  |  |  |  |  |
| Rural | 2701 (79.5) | 54421 (85.5) | 1.00 (Reference) | 1159 (85.3) | 22205 (85.3) | 1.00 (Reference) |
| Central | 697 (20.5) | 9259 (14.5) | 1.76 (1.61-1.94) | 199 (14.7) | 3827 (14.7) | 1.18 (1.00-1.38) |
| **Residential mobilitiy** |  |  |  |  |  |  |
| ≤ 2 residential changes | 1870 (55.0) | 45503 (71.5) | 1.00 (Reference) | 708 (52.1) | 18251 (70.1) | 1.00 (Reference) |
| 3-4 residential changes | 867 (25.5) | 13148 (20.6) | 1.54 (1.41-1.69) | 336 (24.7) | 5527 (21.2) | 1.47 (1.28-1.70) |
| ≥5 residential changes | 661 (19.5) | 5029 (7.9) | 2.19 (1.96-2.43) | 314 (23.1) | 2254 (8.7) | 2.42 (2.07-2.83) |
| **Parent sudden death** |  |  |  |  |  |  |
| No record | 2926 (86.1) | 54464 (85.5) | 1.00 (Reference) | 1153 (84.9) | 21634 (83.1) | 1.00 (Reference) |
| Suicide | 49 (1.4) | 327 (0.5) | 2.03 (1.45-2.86) | 20 (1.5) | 138 (0.5) | 2.14 (1.29-3.53) |
| Other causes | 48 (1.4) | 457 (0.7) | 1.45 (1.05-2.02) | 28 (2.1) | 184 (0.7) | 1.72 (1.11-2.66) |
| No link | 375 (11.0) | 8432 (13.2) | 0.87 (0.72-1.05) | 157 (11.6) | 4076 (15.7) | 0.82 (0.62-1.08) |

^1^Internal comparison, Ors representing added effect of respective variable for those with a registered sick leave
